# The influence of 4G/5G polymorphism in the plasminogen-activator-inhibitor-1 promoter on COVID-19 severity and endothelial dysfunction

**DOI:** 10.3389/fimmu.2024.1445294

**Published:** 2024-08-30

**Authors:** Tetiana Yatsenko, Ricardo Rios, Tatiane Nogueira, Yousef Salama, Satoshi Takahashi, Eisuke Adachi, Yoko Tabe, Nobutaka Hattori, Taro Osada, Toshio Naito, Kazuhisa Takahashi, Koichi Hattori, Beate Heissig

**Affiliations:** ^1^ Department of Research Support Utilizing Bioresource Bank, Graduate School of Medicine, Juntendo University, Tokyo, Japan; ^2^ Department of Enzymes Chemistry and Biochemistry, Palladin Institute of Biochemistry of National Academy of Sciences of Ukraine, Kyiv, Ukraine; ^3^ Institute of Computing, Federal University of Bahia, Salvador, Bahia, Brazil; ^4^ An-Najah Center for Cancer and Stem Cell Research, Faculty of Medicine and Health Sciences, An-Najah National University, Nablus, Palestine; ^5^ Division of Clinical Precision Research Platform, the Institute of Medical Science, the University of Tokyo, Tokyo, Japan; ^6^ Department of Infectious Diseases and Applied Immunology, the Institute of Medical Science, the University of Tokyo, Tokyo, Japan; ^7^ Center for Genome and Regenerative Medicine, Juntendo University, Graduate School of Medicine, Tokyo, Japan; ^8^ Department of Gastroenterology, Juntendo University, Urayasu Hospital, Urayasu-shi, Japan; ^9^ Department of Hematology/Oncology, The Institute of Medical Science, The University of Tokyo, Tokyo, Japan

**Keywords:** plasminogen activator inhibitor-1, endothelial cells, COVID-19, inflammation, PAI-1 polymorphism +43G>A (rs6092), PAI-1 4G/5G promoter polymorphism (rs1799889), plasmin; interleukin-1-β

## Abstract

**Introduction:**

Plasminogen activator inhibitor-1 (PAI-1) is linked to thrombosis and endothelial dysfunction in severe COVID-19. The +43 G>A PAI-1 and 4G/5G promoter polymorphism can influence PAI-1 expression. The 4G5G PAI-1 promoter gene polymorphism constitutes the 4G4G, 4G5G, and 5G5G genotypes. However, the impact of PAI-1 polymorphisms on disease severity or endothelial dysfunction remains unclear.

**Methods:**

Clinical data, sera, and peripheral blood mononuclear cells (PBMCs) of COVID-19 patients were studied.

**Results:**

Comorbidities and clinical biomarkers did not correlate with genotypes in either polymorphism. However, differences between fibrinolytic factors and interleukin-1β (IL-1β) were identified in genotypes of the 4G/5G but not the 43 G>A PAI polymorphism. Patients with the 4G4G genotype of the 4G/5G polymorphism showed high circulating PAI-1, mainly complexed with plasminogen activators, and low IL-1β and plasmin levels, indicating suppressed fibrinolysis. NFκB was upregulated in PBMCs of COVID-19 patients with the 4G4G genotype.

**Discussion:**

Mechanistically, IL-1β enhanced PAI-1 expression in 4G4G endothelial cells, preventing the generation of plasmin and cleavage products like angiostatin, soluble uPAR, and VCAM1. We identified inflammation-induced endothelial dysfunction coupled with fibrinolytic system overactivation as a risk factor for patients with the 5G5G genotype.

## Introduction

Despite the global efforts in vaccination against the coronavirus disease 2019 (COVID-19), the loss of lives due to disrupted coagulation/fibrinolysis and inflammatory response remains a significant concern. The fibrinolytic system counterbalances the coagulation cascade with its leading actor tissue factor (1). During fibrinolysis, plasmin degrades fibrin into fibrin degradation products (FDP), including the clinically meaningful D-dimers, which indicate ongoing thrombosis/fibrinolysis. Why certain patients experience more thrombosis or a more robust inflammatory response/cytokine storm syndrome is not fully understood.

Plasminogen is activated during inflammation (2–5). Tissue- and urokinase-type plasminogen activators (tPA and uPA, respectively) generate plasmin from plasminogen (Plg). Plasmin cleaves uPA and tPA, thereby enhancing their activity. tPA activates plasminogen on fibrin clots. The uPA receptor (uPAR) membrane form activates plasminogen via uPA, promoting cell surface-associated fibrinolysis. uPAR and its soluble fragment s-uPAR generated by proteases like plasmin or uPA exert proinflammatory functions [reviewed in (6)]. Expression of tPA, uPA, PAI-1, and uPAR are upregulated by immune stimuli via transcription factors like NFκB (7, 8).

Plasminogen activator inhibitor-1 (PAI-1; serpine 1) is the principal inhibitor of tPA and uPA [reviewed (2)]. PAI-1 and specific plasmin inhibitor α2-antiplasmin (α2AP) control fibrinolytic activity. Reduced tPA or uPA, increased PAI‐1, or impaired plasminogen levels can establish a hypofibrinolytic state that poses a risk of thrombosis. The suboptimal fibrinolytic response in COVID-19 disease can be attributed to elevated levels of PAI-1, which attenuates plasmin generation and, hence, thrombus dissolution (fibrinolysis) (9).

Plasmin activates cytokines such as transforming growth factor-β (TGFβ) (10–12). Interleukin-1β (13), tumor necrosis factor-α (TNFα), and TGFβ (10) induce *PAI-1* gene expression. TGFβ is secreted in a latent form, and the release of active TGFβ from the complex is catalyzed by proteinases such as plasmin [reviewed in (14)].

PAI-1 biosynthesis can be regulated at the gene level (PAI-1 polymorphisms). The +43G>A polymorphism (rs6092, Ala15Thr) in the first exon of the PAI-1 gene is a missense transition mutation G to A that can change PAI-1 expression. The +43 G polymorphism has been associated with the severity of COVID-19 (15).

The 4G5G PAI-1 promoter gene polymorphism constitutes a common single-base polymorphism (4 vs. 5 consecutive guanine residues) in the promoter region of the PAI-1 gene, 675 base pairs upstream of the transcriptional start site (16). IL-1β and TGFβ modulate the effect of the 4G5G PAI-1 polymorphism on PAI-1 expression. The PAI-1 4G allele produces six times more mRNA than the 5G allele in response to IL-1β. Both 4G and 5G alleles bind a transcriptional activator, such as NFκB, whereas the 5G allele also binds a repressor (17, 18). Subjects homozygous for the 4G allele have plasma PAI-1 concentrations ~25% higher than those homozygous for the 5G allele (17, 18). In this study, we focused on understanding the role of PAI-1 in inflammation-induced endothelial dysfunction.

Here, we investigated the role of the PAI-1 4G/5G and +43G>A polymorphisms on thrombotic and inflammatory response in Japanese COVID-19 patients. Our exploration revealed no discernible genotypic variations in COVID-19 samples concerning the +43G>A polymorphism, possibly due to our study population’s lack of AA genotype. Intriguingly, we pinpointed the 5G5G genotype of the 4G/5G promoter polymorphism as a potential risk factor contributing to inflammation-induced endothelial dysfunction and excessive proteolysis in COVID-19.

## Results

### High circulating PAI-1 in the complicated phase of the disease

The study included COVID-19 Japanese patients with a positive SARS-CoV-2 test and serum and peripheral blood mononuclear cell (PBMC) samples available at diagnosis. The study comprised 35 patients in the uncomplicated phase and 11 patients in the complicated phase. As reported, most patients presented in the uncomplicated phase of the disease (19). Criteria of the complicated phase included intensive care unit admittance and the need for mechanical ventilation, as listed in detail in the Material and Method section. The old age and comorbidities associated with COVID-19 severity, including heart disease, hypertension, and diabetes at diagnosis, indicated a more severe disease (complicated phase; data not shown).

**Figure 1:**
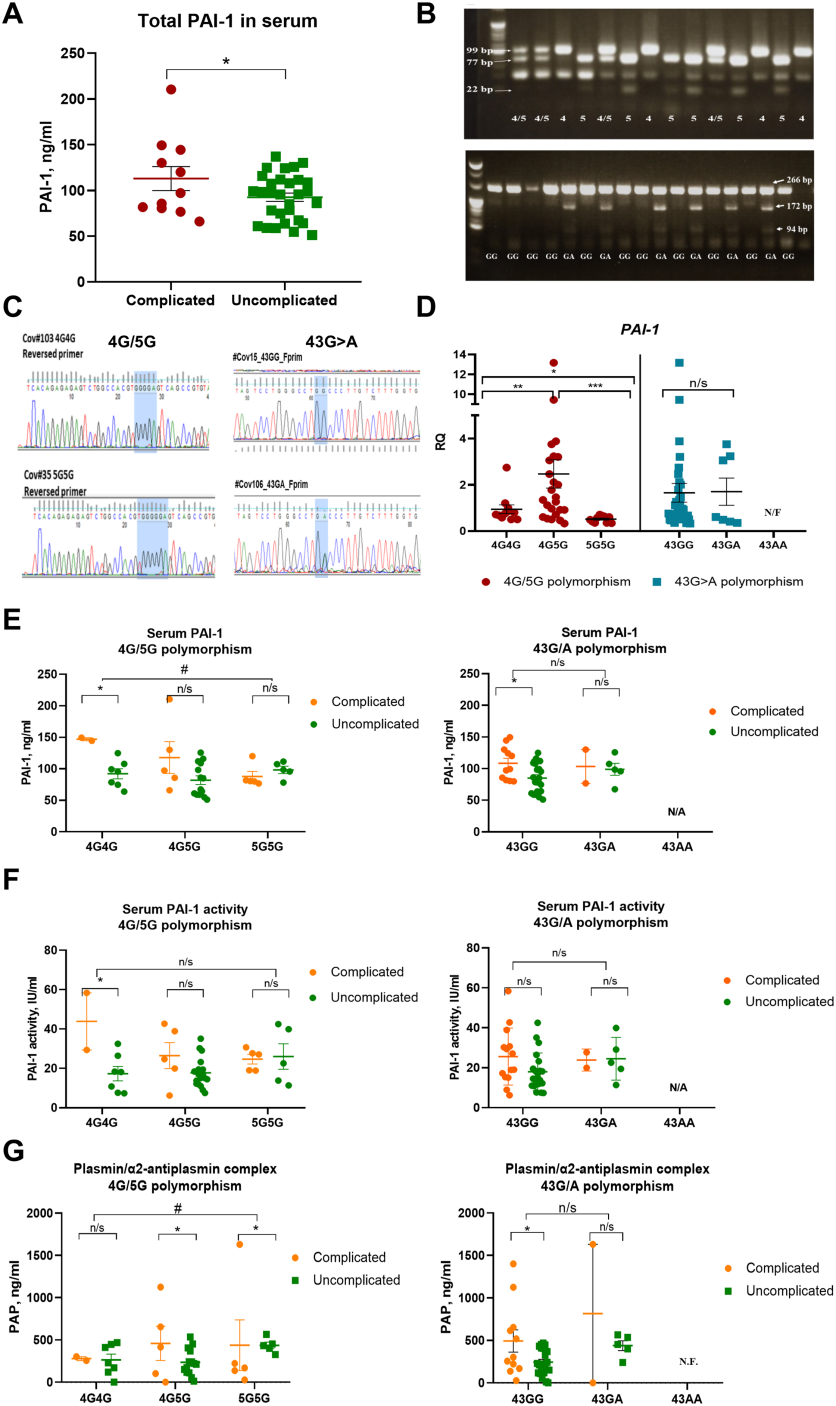
The 4G allele of the PAI-1 promoter is associated with higher PAI-1 transcript and lower plasmin activity in COVID-19 patients. **(A)** PAI-1 levels as measured by ELISA in the study cohort (n=43). **(B)** Polymerase chain reaction-restriction fragment length polymorphism (PCR-RFLP) analyses of all patient samples showed allele-specific fragments after restriction enzyme treatment. **(C)** Examples of Sanger Sequencing for confirmation of the identified PAI-1 4G/5G rs1799889 and +43G>A (rs6092) polymorphisms. In the study group, we did not find the 43AA genotype. Therefore, no data were available (not found, N/F) for this genotype in panels **(D–G)**. **(D)** Fold change in *PAI-1* expression in peripheral mononuclear cells (PBMCs) of COVID-19 patients sorted by genotype (n=42). The expression of indicated genes is normalized to the endogenous reference β-actin and presented as a relative fold change to the expression in 4G/4G groups according to the comparative Ct method (2−ΔΔCt). Experiments were repeated twice with samples run in triplicates each. **(E)** Circulating total PAI-1 protein sorted according to genotypes (data set is the same as in panel **(A)**. **(F)** PAI-1 activity after sorting in genotypes as determined by ELISA [reanalysis of original data published (15)]. **(G)** Serum plasmin/a2-antiplasmin complex levels by ELISA. Dots in each panel represent data from one patient sample. N.F., not found, N/A, not available. Data represent mean ± SEM with p values from unpaired Student’s t-test or the Mann-Whitney test. # p<0.05; * p<0.05; ** p<0.01; *** <0.005; n/s, not significant.

Circulating PAI-1 levels were higher in the complicated phase of the disease (**Figure 1A**). Genotyping results were obtained for the PAI-1 4G/5G promoter (rs1799889) and +43G>A polymorphism (+43G>A (rs6092) using the patients’ PBMCs. **Figure 1B** shows an example of the polymerase chain reaction-restriction fragment length polymorphism (PCR-RFLP) analysis for the PAI-1 polymorphisms. PCR-RFLP results were confirmed by Sanger sequencing (**Figure 1C**). All patients were genotyped, but no mRNA was recovered in one sample.

### COVID-19 comorbidity frequency is similar between genotypes of the 4G/5G PAI-1 promoter and +43G>A polymorphism

Because body weight index (BMI), heart disease, hypertension, and diabetes (20) are comorbidities in COVID-19 but are also linked to the 4G/5G PAI-1 promoter polymorphism, we examined the distribution of these comorbidities among the different genotypes (**Table 1**). Comorbidities were equally distributed among the 4G/5G PAI-1 promoter and +43G>A polymorphism genotypes (**Table 1**).

**Table 1:** Univariate analysis of baseline clinical parameters among Japanese COVID-19 patients at diagnosis.

Total (n=46)	Total (n=46)	4G4G (n=11)	p-values	4G5G (n=25)	p-values	5G5G (n=10)	p-values
**All**	46	11 (23.91%)	–	25 (54.35%)	–	10 (21.74%)	–
**Gender (m/f)**	28/18	6/5 (54.55%/45.45%)	0.8898	14/11 (56.0%/44.0%)	0.6635	8/2 (80.0%/20.0%)	0.2736
**Age**		49.0 [31.5-59.0]	0.4866	55.0 [38.0-67.0]	0.5367	50.5 [33.0-71.75]	0.9894
**BMI**		21.96 [20.69-24.31]	0.2097	24.02 [20.99-26.17]	0.4718	23.78 [22.23-25.81]	0.6601
**Heart (y/n)**	6/40	0/11 (0.0%/100.0%)	0.3113	3/22 (12.0%/88.0%)	1	3/7 (30.0%/70.0%)	0.1066
**Hypertension (y/n)**	15/31	2/9 (18.18%/81.82%)	0.2962	9/16 (36.0%/64.0%)	0.8262	4/6 (40.0%/60.0%)	0.7065
**Diabetes (y/n)**	8/38	2/9 (18.18%/81.82%)	1	3/22 (12.0%/88.0%)	0.4390	3/7 (30.0%/70.0%)	0.3442
Total (n=46)	Total (n=46)	43 GG	43 GA	p-values	43 AA	p-values	
**All**	46	39 (84.78%)	7 (15.22%)	–	–	–	
**Gender (m/f)**	28/18	25/14 (64.1%/35.9%)	3/4 (42.86%/57.14%)	0.4069	–	–	
**Age**		51.0 [35.0-65.5]	62.0 [36.0-70.0]	0.8304	–	–	
**BMI**		23.72 [20.78-25.6]	24.37 [22.4-26.05]	0.7324	–	–	
**Heart (y/n)**	6/40	4/35 (10.26%/89.74%)	2/5 (28.57%/71.43%)	0.2214	–	–	
**Hypertension (y/n)**	15/31	13/26 (33.33%/66.67%)	2/5 (28.57%/71.43%)	1	–	–	
**Diabetes (y/n)**	8/38	6/33 (15.38%/84.62%)	2/5 (28.57%/71.43%)	0.5874	–	–	

Patients showing one speciﬁed comorbidity alone or a combination were enrolled in the yes group. Data are n (%). BMI, body mass index; heart, patients with underlying heart disease. P-values were calculated as appropriate by X^2^ or Fischer’s exact test. Data were reanalyzed from a more extensive study cohort (15).

### High TroponinT levels in 5G5G of the 4G/5G PAI-1 promoter, but not in genotypes of the +43G>A polymorphism

Correlation analyses were performed with markers linked to thrombosis (D-dimer, fibrinogen, platelet counts, prothrombin time (PT), or TroponinT), and inflammation (white blood cell counts, lymphopenia with low lymphocyte counts, and C-reactive protein (CRP), interleukin-6 or procalcitonin levels) to identify associations between PAI-1 polymorphisms and thrombosis- or inflammation-associated clinical parameters in COVID-19 patients. No significant associations were found for thrombosis- and inflammation-associated markers between the 4G4G and 5G5G groups and +43G>A polymorphism (**Table 2**). Levels for Troponin T, a marker associated with disease severity (21) and myocardial infarction, were higher in the 5G5G compared to the 4G5G (p=0.0278) and borderline higher compared to the 4G4G genotype (p=0.0893; **Table 2**).

**Table 2:** Clinical inflammation- and thrombosis-associated biomarkers sorted according to COVID-19 patients` genotypes of the 4G/5G and + >43 G PAI-1 gene polymorphisms.

Thrombosis-associated marker	4G4G (n=11)	4G5G (n=10)	p-values	4G4G (n=11)	5G5G (n=10)	p-values	GG (n=39)	GA (n=7)	p-values
D-Dimer (0-1µg/dL)	1.75 [1.3-2.08]	1.5 [1.2-1.8]	0.4976	1.75 [1.3-2.08]	1.9 [1.35-2.6]	0.8199	1.65 [1.23-2.0]	1.9 [1.25-8.9]	0.3542
Fibrinogen (150 - 400mg/dL)	418.0 [363.5-504.0]	402.0 [358.5-510.5]	0.9070	418.0 [363.5-504.0]	381.0 [349.25-591.5]	0.8461	453.5 [376.0-577.0]	358.0 [311.5-388.0]	0.2409
Platelets (153-346x103/µL)	207.0 [166.0-228.75]	200.0 [170.0-220.0]	0.6219	207.0 [166.0-228.75]	203.5 [173.75-223.25]	1	196.0 [166.0-226.0]	204.0 [187.5-220.5]	0.6954
PT INR	0.99 [0.98-1.0]	0.99 [0.96-1.04]	0.7594	0.99 [0.98-1.0]	1.0 [0.95-1.07]	0.8083	0.99 [0.96-1.04]	1.0 [0.95-1.1]	1
Troponin T(0-0.1ng/dL)	3.5 [0.76-4.0]	5.0 [4.5-10.0]	0.0278	3.5 [0.76-4.0]	8.0 [3.0-12.25]	0.0893	5.0 [3.0-8.75]	5.0 [4.0-11.0]	0.5043
Inflammation-associated marker									
WBC (3.9-9.7 x10^3^/µL)	4.85 [3.88-5.58]	4.5 [3.6-5.9]	0.9417	4.85 [3.88-5.58]	4.65 [4.32-6.1]	0.7623	4.85 [3.8-5.98]	4.2 [3.7-4.4]	0.3393
Neutrophil (25-60 x10^2^/µL)	3.31 [2.69-828.89]	3.0 [2.24-4.51]	0.3518	3.31 [2.69-828.89]	4.46 [1.9-8.76]	0.7337	3.34 [2.43-6.19]	2.59 [1.87-4.68]	0.3161
Lymphocytes (x10²/µL)	28.95 [20.98-30.4]	30.5 [20.4-37.0]	0.4764	28.95 [20.98-30.4]	20.8 [12.78-34.75]	0.6775	28.55 [19.27-35.12]	32.2 [16.85-39.45]	0.6954
CRP (0-0.29mg/dL)	0.66 [0.24-3.19]	0.65 [0.15-4.31]	0.8408	0.66 [0.24-3.19]	0.34 [0.1-5.9]	0.7622	0.66 [0.15-4.2]	0.18 [0.14-3.42]	0.6497
IL6 (0-4pg/mL)	3.9 [2.8-7.1]	4.9 [2.1-18.15]	0.6149	3.9 [2.8-7.1]	4.1 [1.6-5.6]	0.7572	4.6 [1.8-12.4]	4.1 [2.35-6.0]	0.8083
Procalcitonin(0-0.04ng/dL)	0.06 [0.04-0.08]	0.05 [0.03-0.07]	0.7544	0.06 [0.04-0.08]	0.04 [0.03-0.06]	0.4019	0.05 [0.03-0.08]	0.04 [0.03-0.06]	0.4035

Data are means [range] of absolute values per patient obtained at diagnosis. P-values were obtained by comparing the values of a biomarker, considering the genotype, and calculated by X^2^ or Fischer’s exact test. PT INR, expressed as International Normalized Ratio; prothrombin; WBC, white blood cell counts; IL6, interleukin-6; CRP, C-reactive protein. The data presented here were part of a more extensive study and were analyzed according to patient genotypes [see (14)].

### Genotype frequencies of two PAI-1 polymorphisms of COVID-19 patients in the uncomplicated and complicated phase

Most patients were in the uncomplicated phase at diagnosis independent of their PAI-1 genotype. When all patients independent of the clinical phase were considered, the allele frequencies for 4G and 5G were identical (**Table 3**). However, the 5G allele and 5G homozygosity were more frequent in patients in the complicated phase of COVID-19 (**Table 3**).

**Table 3:** Distribution of PAI-1 genotype frequencies in COVID-19 patients in the uncomplicated and complicated phase.

Genotypes	All (n=46)	Uncomplicated (n=35)	Complicated (n=11)	OR (95% CI)
4G/5G PAI-1 promoter polymorphisms				
**4G/4G**	11	9 (81.82%)	2 (18.18%)	1.54 (0.24-17.34)
**4G/5G PAI-1 promoter polymorphisms**	25	20 (80.0%)	5 (20.0%)	1.58 (0.33-7.95)
**5G/5G**	10	6 (60.0%)	4 (40.0%)	2.69 (0.44-15.54)
Allelic frequency				
**4G**	47 (51.09%)	38.0 (80.85%)	9.0 (19.15%)	1.71 (0.59-5.16)
**5G**	45 (48.91%)	32.0 (71.11%)	13.0 (28.89%)	
43 G PAI-1 promoter polymorphisms				
**GG**	39	30 (76.92%)	9 (23.08%)	1.32 (0.11-9.98)
**GA**	7	5 (71.43%)	2 (28.57%)	
**AA**	0	0	0	
Allelic frequency				
**G**	85 (92.39%)	65.0 (76.47%)	20.0 (23.53%)	1.30 (0.12 - 8.69)
**A**	7 (7.61%)	5.0 (71.43%)	2.0 (28.57%)	

Patients showing one speciﬁed genotype were enrolled in the yes group. Data are n (%). The odds ratio (OR) measures the relative risk of COVID-19 infection between subjects with the 4G/4G genotype and subjects with the 4G/5G or 5G/5G genotype.

The PAI-1 genotype frequencies of the +43G>A polymorphism showed a dominance of the 43GG and 43GA genotypes and no cases of the 43AA genotype in the studied cohort (**Table 3**). A similar GG and GA genotype frequency distribution was found in patients in the uncomplicated and complicated phase. Our patient cohort did not have patients in the uncomplicated and complicated phase carrying the A allele of +43G>A polymorphism.

### Lowest PAI-1 transcripts in PBMCs of COVID-19 patients with the 5G5G genotype

Circulating PAI-1 levels in patient blood samples were higher in the complicated than the uncomplicated phase, as measured by ELISA and recently reported (15) (**Figure 1A**). Polymorphism analysis was determined by polymerase chain reaction-restriction fragment length polymorphism (PCR-RFLP; **Figure 1B**) analysis followed by Sanger Sequencing (**Figure 1C**). PAI-1 transcript levels in PBMCs differed among genotypes for the PAI-1 4G/5G but not the +43G>A polymorphism (**Figure 1D**). Overall, the highest *PAI-1* expression was found among the 4G5G, followed by the 4G4G genotypes, and the lowest transcript levels in the 5G5G genotypes (**Figure 1D**). These data suggested that the 5G5G genotype is associated with the lowest PAI-1 transcript levels in PBMCs.

### 4G4G COVID-19 patients show high PAI-1 and low plasmin levels

PAI’s effect on plasmin activity, the critical mediator of clot dissolution, is mediated by inhibiting plasminogen activators. Plasminogen concentration in blood is relatively high (around 180 µg/ml), and only a small part is converted to plasmin during fibrinolysis, which is immediately after blood clot dissociation is inhibited by α2-antiplasmin and other minor inhibitors generating the plasmin/inhibitor complexes such as plasmin/α2-antiplasmin (Pm/α2AP; PAP). The plasmin/α2-antiplasmin complex represents plasmin formation in the last 2-8 hours (22).

No differences were evident between genotypes of the +43G>A polymorphism for total PAI-1 protein and activity (based on data reanalyzed from a published data set (15)) and PAP complexes in the uncomplicated phase (**Figures 1E, F**). Because of the low A allele frequency of the 43G/A polymorphism in our data set, which leads to low patient numbers, especially in the complicated phase, we cannot exclude that this polymorphism might affect PAI-1 or PAP levels. Given these limitations of our sample distribution, we focused further analysis on the 4G/5G polymorphism, which shows the following genotypes: 4G4G, 4G5G, and 5G5G.

In the uncomplicated phase, circulating total PAI-1 protein, active PAI-1, and PAP serum levels were similar between genotypes of the 4G/5G polymorphism (**Figures 1E–G**). In the complicated phase, there was a trend towards more total PAI-1 protein and active PAI-1 in sera of patients with the 4G4G genotype of the 4G/5G polymorphism than the 5G5G genotype (**Figures 1E, F**).

In the studied cohort, the total PAI-1 antigen level was higher than reported for healthy donors (up to 46 ng/ml according to Mosby’s manual of diagnostic and laboratory tests (23)). Interestingly, the levels of active PAI-1 were below the upper limit of the normal range (32 IU/ml) in most patient samples. Our previous studies demonstrated that Japanese COVID-19 patients have lower risks of thrombo-inflammation than Germans (19), and Japanese healthy controls had lower PAI-1 activity than Europeans (15). The difference in PAI-1 protein level and its activity may explain the low immuno-thrombosis susceptibility of the Japanese population.

PAP levels did not change in the 4G4G group but increased in the 4G5G and 5G5G genotypes in the complicated phase (**Figure 1G**). These data suggested differences in PAI-1 expression among genotypes of the 4G/5G polymorphism might be linked to severe inflammation (the complicated phase of the disease). However, patient numbers in the complicated phase were low.

### Higher *NFκB* and *KLF2* expression in PBMCs of COVID-19 patients with the 4G4G genotype

PAI-1 and plasmin levels differed among 4G/5G genotypes, and a trend towards higher total PAI-1 and plasmin levels was observed mainly in the complicated clinical phase with robust inflammatory response and cytokine elevation. These results lead us to speculate that the observed differences in plasmin activation between the 4G4G versus 5G5G patients (PAI-1 4G/5G polymorphism) are linked to inflammation (18).

**Figure 2:**
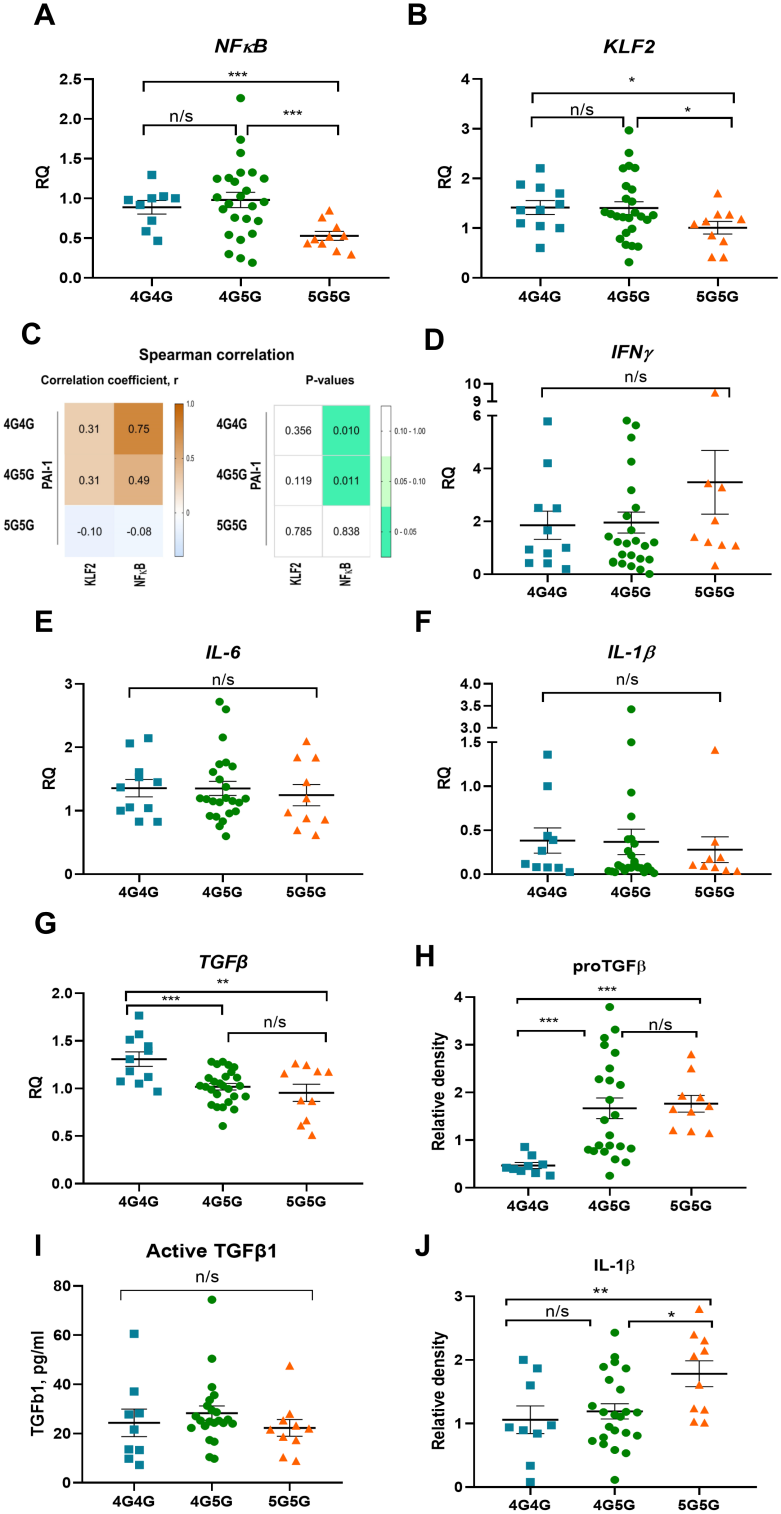
Circulating proTGFβ and IL-1β protein but not cytokine transcripts in PBMC of COVID-19 patients were higher in 5G5G patients. *NFkB* **(A)** and *KLF2* **(B)** expression in PBMCs of COVID-19 patients were sorted for genotypes of the 4G5G PAI-1 promoter polymorphism. **(C)** Heat map of Spearman’s correlation coefficient between 4G5G PAI-1 polymorphism coefficient and the transcription factors NFκB and KLF2 in COVID-19 patients. The correlation coefficients are represented in the white-brown color intensity change (left panel), as shown in the color bar. P-values of the parameter correlations are given using white-green color coding (right panel). P values were statistically significant (p<0.05) with 95% CIs for each correlation coefficient. **(D–G)** Fold change in *IFNg* **(D)**, *IL-6* **(E)**, *IL-1b* **(F)**, and *TGFβ* **(G)**, expression in PBMCs of COVID-19 patients sorted for genotypes of 4G5G PAI-1 promoter polymorphism. The expression of the indicated genes is normalized to the endogenous reference β-actin and presented as a relative fold change to expression in 4G/4G groups according to the comparative Ct method (2−ΔΔCt). Gene expression data: Experiments were repeated twice using at least 2-3 samples per test group. **(H, I)** Analysis of proTGFβ **(H)** by western blotting, active TGFβ by ELISA **(I)**, and IL-1β by western blotting **(J)** in sera of COVID-19 patients. **(H, I)** Western data are quantified by band intensity data normalized to a sample from a healthy donor and are the reanalysis of original data published in (15). Data represent mean ± SEM. Depending on the sample normality, p values were determined using the unpaired Student’s t-test or the Mann-Whitney test. * p<0.05; ** p<0.01; *** <0.005; n/s, not significant.

The 4G and 5G alleles bind a transcription factor like NFκB. The 5G allele, in addition, can bind a repressor, resulting in a dampened NFκB response and lower PAI-1 levels (17, 18). High *NFκB* expression was found in COVID-19 patient’s PBMCs of the 4G4G and 4G5G but not 5G5G genotypes with significant differences between 4G4G and 5G5G groups (**Figure 2A**). The Krüppel-like factor 2 (KLF2) transcription factor modulates PAI-1 expression in ECs (24). *KLF2* expression was higher in PBMCs of the 4G4G than in the 5G5G genotypes (**Figure 2B**). Spearman analysis revealed NFκB, but not KLF2, correlated with PAI-1 expression in PBMCs with the 4G4G and 4G5G but not the 5G5G genotypes (**Figure 2C**). These data indicated the proinflammatory transcription factor NFκB positively associated with the 4G4G and 4G5G, but not the 5G5G genotypes.

### Low IL-1β serum levels and high NFκB transcripts in PBMCs in COVID-19 patients with 4G4G genotype

Because NFκB positively correlated with the 4G4G and 4G5G genotypes in PBMCs, the NFκB cytokine target genes IL-1β, IL6, interferon-gamma (IFNγ), and TGFβ were determined. Despite high *NFκB* transcripts in 4G4G and 4G5G PBMCs, *IFNγ*, *IL-6*, and *IL-1β* expression was similar between tested genotypes (**Figures 2D–F**). *TGFβ* expression was higher in 4G4G than in 4G5G or 5G5G genotype PBMCs (**Figure 2G**). A similar cytokine and transcription factor expression trend was found after subgrouping cytokine expression data according to the patient’s clinical phase (data not shown). Despite low proTGFβ serum levels in 4G4G genotypes (**Figure 2H**), active TGFβ serum levels were similar between the 4G/5G genotypes (**Figure 2I**). Although *TGFβ* expression was higher in 4G4G PBMCs, active TGFβ1, whose generation requires plasmin activation, was not different.

Despite similar transcript levels in PBMCs, circulating IL-1β levels were lowest in the 4G4G and highest in the 5G5G genotype and different between the genotypes (**Figure 2J**). No transcription and cytokine expression differences were seen in the +43 G genotypes (data not shown). These results indicated that COVID-19 patients with the 4G4G genotype had the highest *NFκB* expression in PBMCs and the lowest circulating IL-1β levels.

### Shutdown of fibrinolysis in IL-1β-stimulated 4G4G ECs

**Figure 3:**
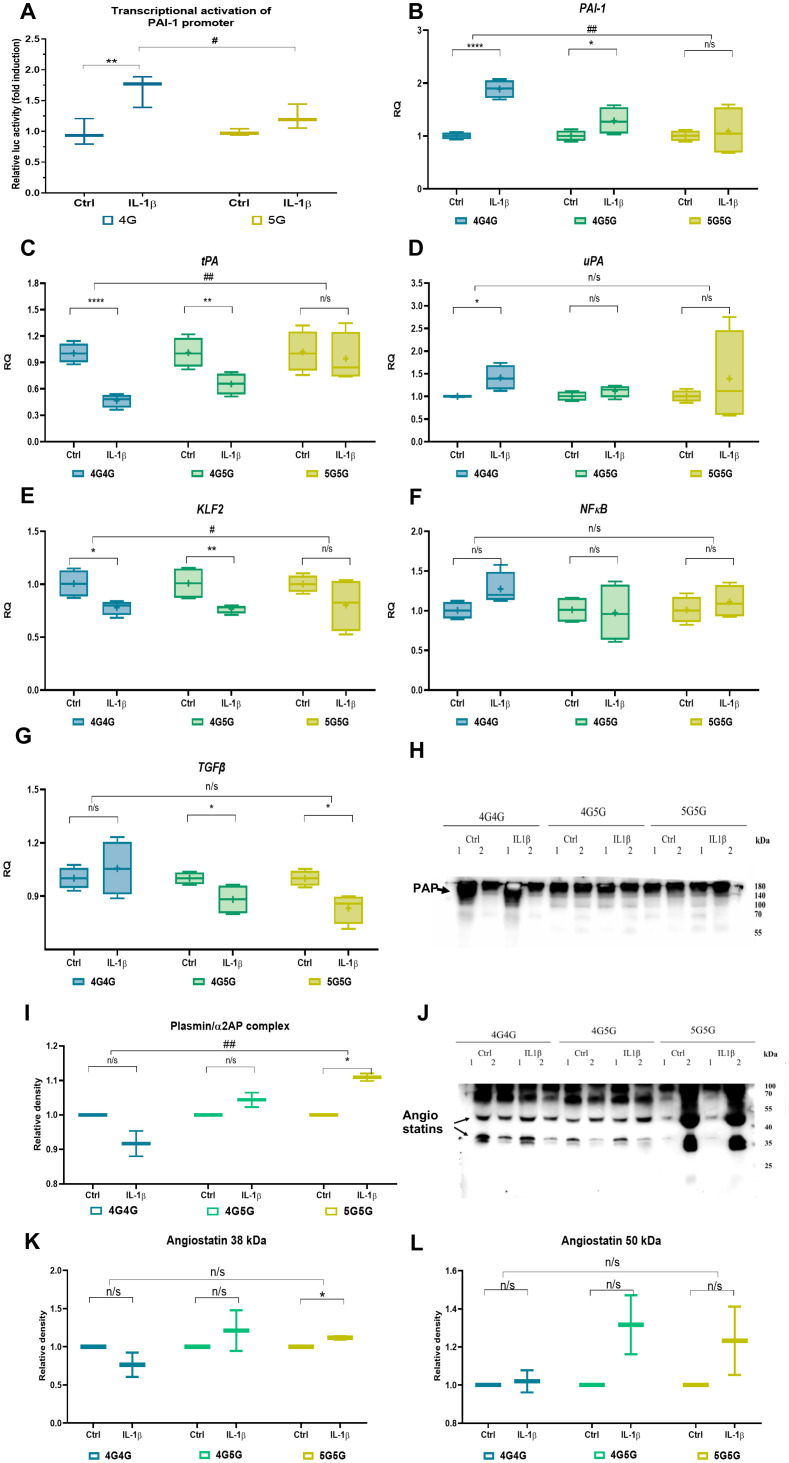
IL-1β establishes an anti-fibrinolytic gene expression profile in 4G4G primary endothelial cells (ECs). **(A)** Transcriptional activation of the human PAI-1 promoter by recIL-1β. HUVEC cells were transiently transfected with the PAI-1 4G and 5G luciferase reporter plasmids. Cells were treated with or without IL-1β for 24 hours before lysis. Firefly luciferase activity was normalized to Renilla luciferase activity and is expressed as fold change to controls. Data shown are the mean ± SEM of triplicates from a representative experiment (n=3/group). **(B–G)** Fold change in *PAI-1* **(B)**, *tPA* **(C)**, and *uPA* **(D)**, *KLF2* **(E)**, *NFκB* **(F)**, and *TGFb* **(G)** expression in cultured 4G4G, 4G5G, or 5G5G ECs stimulated with rec IL-1β (two independent experiments using two different cell origins per genotype were performed; data shown are from one experiment; n=4/group). The expression of the indicated genes is normalized to the endogenous reference β-actin and presented as a relative fold change to expression in the control expression of each genotype according to the comparative Ct method (2−ΔΔCt). **(H)** Immunoblot of plasmin/α2-antiplasmin complex (PAP) in an equal volume of supernatants of EC cultures. **(I)** Band intensity quantified of the G blot, whereby each recIL-1β sample was normalized to its control samples. **(J–L)** A representative immunoblot of Plg using supernatants from cultures treated with or without (control) IL-1β showed angiostatin fragments **(J)** after loading an equal volume of supernatants from EC cultures. Band intensity quantified of the I blot, whereby each recIL-1β sample was normalized to its control samples, showing the Plg cleavage fragment angiostatin at 38 kDa **(K)** and 50 kDa **(L)**. All western blots were performed at least twice with similar results. # p<0.05; ## p<0.01; * p<0.05; ** p<0.01; *** p<0.005; n/s, not significant. *In vitro* data were presented as box plots to discriminate *in vivo* data from the following *in vitro* data. All experiments were done in triplicate, and two cell lines for each genotype were used.

The data on more patients in the 5G5G group with higher Troponin T and being found in the complicated phase might hint at endothelial dysfunction during COVID-19 infection. Endothelial dysfunction has been reported in COVID-19 (15, 25). Infection-induced IL-1β activation contributes to the impaired endothelial barrier function and procoagulant/anti-fibrinolytic shift in the endothelium (26). Previous studies have shown that the 4G/5G locus present in the PAI-1 promoter is required for IL-1β-induced PAI-1 gene expression in HepG2 cells (18). To better understand whether IL-1β can activate PAI-1 gene expression through the 4G or 5G locus in endothelial cells (ECs), we examined the effect of IL-1β on the PAI-1 4G or 5G promoter transcriptional activity using the reporter vector pGL4.1-[luc2] where the 4G or 5G promotor sequences were placed upstream of the luciferase gene. Transient transfection of these reporters into human umbilical vein endothelial cells (HUVEC) cells resulted in similar basic transcription activities, which were increased in response to recombinant IL-1β after overnight incubation in HUVEC cells transfected with the 4G4G, but not the 5G5G PAI-1 promoter plasmid (**Figure 3A**). These data suggest that the 4G4G region of the PAI-1 promoter is required for IL-1β-mediated PAI-1 induction.

To further investigate the influence of the 4G5G polymorphism on IL-1β-mediated fibrinolysis-associated factor production, primary ECs (human lung microvascular endothelial cells and and HUVECs) with known genotypes (two EC sources per genotype) were incubated with recombinant IL-1β overnight.

IL-1β treatment enhanced *PAI-1* and *uPA* and downregulated *tPA* expression in 4G4G and 4G5G but not 5G5G genotypes (**Figures 3B–D**). *PAI-1* and *tPA*, but not *uPA* expression, differed between 4G4G and 5G5G genotypes. Given that the primary plasminogen activator produced ECs is tPA, these data indicated that IL-1β in the 4G4G genotype suppresses fibrinolytic factor expression in ECs, indicative of a fibrinolytic shutdown.

IL-1β suppresses KLF2 expression in HUVECs (27). IL-1β downregulated *KLF2*, not *NFκB* expression in 4G4G and 4G5G but not the 5G5G genotypes (**Figures 3E, F**). However, IL-1β impaired *TGFβ* expression in ECs with 4G5G and 5G5G, not 4G4G genotypes (**Figure 3G**).

### Protease cleavage products in supernatants of IL-1β−stimulated 5G5G ECs

More plasmin (PAP) was found in IL-1β-stimulated EC cultures of the 5G5G genotype than in the 4G4G genotype, indicating enhanced plasmin proteolytic activity (**Figures 3H, I**).

Plasmin generates plasminogen (Plg) fragments, such as the kringle-containing angiostatin K1-3 (38 kDa), K1-4 (50 kDa), and K1-5 (56 kDa) (28) and converts pro-forms of matrix metalloproteinases (MMPs) into their active forms (2, 4, 29, 30). IL-1β-stimulated ECs released more of the Plg fragment 38 kDa (K1-3) but not 50 kDa (K1-4) angiostatin into 5G5G, but not 4G4G supernatants (**Figures 3J–L**). 

K1-3 (38 kDa) can be generated by neutrophil elastase (31) and MMP7. Neutrophil elastase is mainly expressed in neutrophils and not EC. We, therefore, analyzed MMP7 expression in culture supernatants. MMP7 protein was induced in IL-1β-stimulated EC cultures of 4G5G cells (data not shown). In summary, PAI-1 elevation after IL-1β did not occur in the 5G5G ECs, establishing a plasmin-mediated proteolytic status/niche.

### IL-1β-stimulated ECs form PA/PAI-1 complexes, resulting in a fibrinolysis shutdown

**Figure 4:**
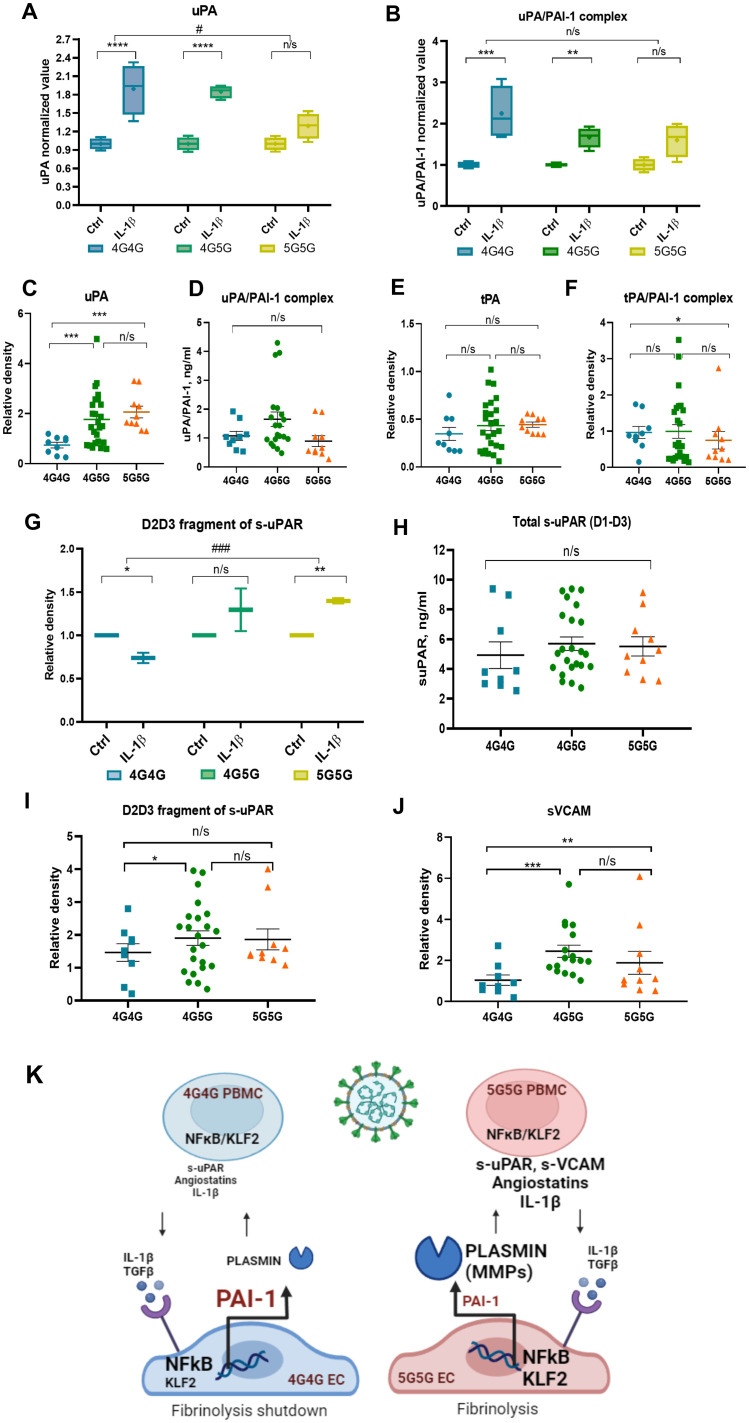
The 4G4G genotype associates with high tPA/PAI-1 complexes and lower proteolytic fragments angiostatin 38kDa, s-uPAR, and sVACM1 in COVID-19 patient sera and rec IL-1β stimulated ECs. **(A, B)** ECs with known 4G5G PAI-1 promoter genotypes were cultured overnight with IL-1β. A representative immunoblot of uPA **(A)** and uPA/PAI-1 complex **(B)** and band intensity quantification after loading an equal volume of supernatants from EC cultures. **(C)** Free/uncomplexed uPA [**(C)**; a reanalysis of original data published in (15)] after band quantification of an immunoblot in COVID-19 sera. Serum uPA/PAI-1 complex **(D)** levels measured by ELISA in COVID-19 patients with indicated genotypes. **(E, F)** Free/uncomplexed tPA **(E)** and tPA/PAI-1 complex **(F)** levels were determined by western blotting [data were analyzed according to genotypes: original data in (15)]. **(G)** Supernatants collected from rec IL-1β-and carrier/control-treated EC cultures were analyzed by western blot analysis for the D2D3 fragment of s-uPAR. Band intensity was quantified by normalizing each rec IL-1β sample to its control sample. **(H)** Total s-uPAR (D1-D3) in COVID-19 sera as determined by ELISA. **(I, J)** Western blot analysis for s-uPAR/D2D3 **(I)** and sVCAM **(J)** in sera of COVID-19 patients sorted according to their genotypes for the 4G5G PAI-1 Promoter polymorphism [reanalysis of original data in (15)]. **(K)** Proposed model for the influence of the 4G5G polymorphism during SARS-CoV-2 infection with a robust inflammatory response: Higher IL-1β was found in the circulation of COVID-19 patients with the 5G5G genotype. IL-1β upregulates *PAI-1* and *NFκB* expression in ECs with the 4G4G but not the 5G5G genotype. Cytokine (IL-1β)-induced PAI-1 forms complexes with its activators tPA and uPA, especially in the 4G4G genotype, resulting in impaired uPA and tPA-driven plasmin generation in the 4G4G genotype, ultimately generating a proteolytic shutdown. In ECs, IL-1β downregulates KLF2 expression, possibly to counterbalance PAI-1 upregulation. This process is the opposite in 5G5G EC. Low PAI-1 levels after IL-1β stimulation contribute to high active plasmin that further fuels IL-1β and TGFβ generation and establishes a proteolytic niche. *In vitro* data were presented as box plots to discriminate *in vivo* data from the following *in vitro* data. All experiments were done in triplicate, and two cell lines for each genotype were used. NFκB, Nuclear factor kappa-light-chain-enhancer of activated B cells; KLF2, Krüppel-like factor 2; PBMC, peripheral blood mononuclear cells; interleukin-1 β, IL-1β, TGF β, transforming growth factor-β; MMP, matrix metalloproteinase. Data represent mean ± SEM. Depending on the sample normality, p values were determined using the unpaired Student’s t-test or the Mann-Whitney test. * p<0.05; **p<0.01; *** p<0.001; n/s, not significant.

We reported low circulating uPA and uPA/PAI-1 complexes in severely ill COVID-19 patients (15). IL-1β enhanced uPA release from ECs (**Figure 4A**), which was mainly complexed with PAI-1 forming uPA/PAI-1 complexes in the 4G4G and 4G5G but not 5G5G genotypes (**Figures 4A, B**).

The analysis of COVID-19 sera revealed that free, uncomplexed uPA was highest in the sera of patients with the 5G5G genotype (**Figure 4C**). No differences between genotypes were found in uPA/PAI-1 complex serum levels (**Figure 4D**). Free, non-complexed tPA levels were similar between genotypes (**Figure 4E**), but fewer circulating tPA/PAI-1 complex levels were found in 5G5G compared to 4G4G sera (**Figure 4F**). Our data suggested that COVID-19-/IL-1β-driven PAI-1 and plasminogen activator (PA) upregulation enhanced PA/PAI-1 complex formation in the 4G4G genotype, trapping PAs and causing a fibrinolysis shutdown. In contrast, due to impaired PAI-1 induction in the 5G5G genotype, IL-1β-/COVID-19-induced retained free PA establish a profibrinolytic state.

### Increased release of s-uPAR from IL-1β-stimulated ECs with the 5G5G genotype

uPAR discriminated between COVID-19 patients who might or might not benefit from IL-1 blockade treatment (32). Plasmin converts pro-uPA into active uPA (33). Active uPA cleaves membrane uPAR, releasing D1-D3 (full-length extracellular molecule) and D2D3 fragments (size ~40kDa) (34). In line with high uPA and plasmin generation in human ECs cultured *in vitro* with rec.IL-1β, more D2D3 fragments were found in IL-1β-stimulated 5G5G supernatants but not 4G4G ECs (**Figure 4G**).

To confirm our *in vitro* observation of IL-1β stimulation of HUVEC cells, we analyzed s-uPAR and its D2D3 fragments in patient sera. The analysis of circulating total s-uPAR (D1-D3) in COVID-19 patients as determined by ELISA showed no significant difference between genotypes (**Figure 4H**). However, D2D3 fragment serum levels were higher in 4G5G compared to 4G4G genotypes (**Figure 4I**).

Vascular cell adhesion molecule 1 (VCAM1) is an adhesion molecule expressed on ECs that controls cell adhesion and the inflammatory response during SARS-CoV-2 infection (35). Like s-uPAR, proteases can release VCAM1 into circulation as soluble VCAM1. In line with protease activation (“proteolytic niche”), more sVCAM1 was detected in the sera of patients with the 5G5G than in the 4G4G genotype (**Figure 4J**).

## Discussion

Despite widespread vaccination efforts globally, COVID-19 continues to claim lives, partly due to dysregulated coagulation/fibrinolysis and inflammatory responses (36). Analyzing PAI-1 polymorphism as a genetic factor that regulates fibrinolysis could predict life-threatening thrombosis, which could save lives.

We show that the 4G/5G PAI-1 promoter polymorphism is a critical determinant of fibrinolytic and proteolytic activity on EC surfaces after COVID-19 infection and is linked to IL-1β increases. Still, when IL-1β is low or absent, the 4G/5G PAI-1 promoter polymorphism does not influence fibrinolytic and proteolytic activity.

Our data suggest that the 4G/5G PAI-1 promoter polymorphism controls PAI-1 induction and the fibrinolytic and proteolytic response under inflammatory pressure in ECs. The 4G4G genotype is endowed with impaired fibrinolytic and proteolytic activity due to IL-1β-mediated PAI-1 upregulation, resulting in an increased thrombotic risk (**Figure 4K**).

We tested the +43 G>A PAI-1 polymorphism and 4G/5G PAI-1 promoter polymorphism in Japanese COVID-19 patients. Our data on the Japanese COVID-19 patients demonstrated – in contrast to the study with Mexican COVID-19 patients (37) – that total PAI-1 and plasmin levels differed between genotypes of the 4G/5G, but not the +43 G>A PAI-1 polymorphism in COVID-19 patients. Japanese COVID-19 patients with the 4G4G genotype had higher PAI-1 and lower plasmin levels, establishing a fibrinolytic shutdown and prothrombotic condition. In support of our findings, the 4G4G genotype in a large meta-analysis was associated with an increased venous thromboembolism risk among Asia populations (38).

We did not further analyze the +43G>A polymorphism as we did not find a single patient with the AA genotype. Our data are consistent with a study cohort of 227 healthy Asian women, where no AA genotype was detected (39), supporting our findings that the AA genotype is extremely rare in Asians.

We confirm reports by others (18) showing *PAI-1* expression was independent of the patient’s genotype for the 4G/5G polymorphism in the uncomplicated phase. The differential response between genotypes for the 4G/5G polymorphism became apparent in patients with high IL-1β levels, a typical cytokine that indicates severe inflammation and an inflammatory response (40). IL-1β is a critical switch on ECs to control local fibrinolysis mediated by the 4G/5G PAI-1 polymorphism. Mechanistically, IL-1β enhanced *PAI-1* expression, promoted PAI-1 complex formation with tPA or uPA, and reduced *tPA* expression, establishing fibrinolytic shutdown in 4G4G ECs. The lack of PAI-1 response and unchanged tPA and uPA expression after IL-1β stimulation in 5G5G ECs creates a hyperfibrinolytic state with plasmin activation (proteolytic niche) (**Figure 4M**). This proteolytic storm enabled the cleavage of membrane proteins like uPAR and circulating proteins like Plg, leading to enhanced s-uPAR and Plg fragment K1-3 (angiostatin) formation.

Why this study – using Japanese patient samples – but not other studies could identify the 4G/5G PAI-1 polymorphism as critical for the fibrinolytic response in COVID-19 patients, it is essential to see the differences in the study groups. First, compared to other Western countries, the 4G4G genotype is more frequent in Japanese (41), making differences between the 4G4G and 5G5G genotypes easier to detect. Second, obesity is not so prevalent in Japan, although it is a pandemic worldwide. Metabolic syndrome-associated diseases like adiposity, diabetes, and hypertension, even without COVID-19, can establish chronic low-level inflammation that alters PAI-1 expression (37, 42, 43). These two conditions possibly contributed to identifying links between COVID-19 and the 4G/5G PAI-1 polymorphism. In the clinical samples of COVID-19 patients, we cannot rule out that subjects’ comorbidity profiles might modulate PAI-1 expression. A recent meta-analysis showed that the PAI-1 4G/5G polymorphism was associated with coronary artery disease risk (44). We found higher Troponin T levels – associated with heart disease – in 5G5G COVID-19 patients.

Even though plasma PAI-1 circulates at low levels (5-50ng/ml), it mainly adopts the active conformation. In our study the total antigen level of PAI-1 was higher in COVID-19 patients compared to the normal range (23). Surprisingly, PAI-1 activity levels were below the known upper limits of the normal range, suggesting that most PAI-1 is inactive. 

Our previous studies demonstrated that Japanese COVID-19 patients are less likely to develop thrombo-inflammation than Germans. Japanese healthy controls have lower PAI-1 activity than Europeans (15). The differences in PAI-1 protein level and activity may explain the low immune-thrombosis susceptibility in the Japanese population.

We found that COVID-19 patients with the 4G4G genotype had high *PAI-1* transcript levels in PBMCs and low circulating plasmin and IL-1β or proTGFβ levels, indicating a mild inflammatory response. We found a strong positive correlation of *NFκB* with the 4G4G and 4G5G genotypes, which might have contributed to the high *TGFβ* expression levels in 4G4G patients. While the higher *TGFβ* transcripts might be linked to higher NFκB expression in 4G4G PBMCs, it is unclear why low circulating proTGFβ was found in this genotype. Active TGFβ was similar between 4G5G genotypes, making its influence on PAI-1 expression less likely. The lack of plasmin and possibly other protease generation in the 4G4G genotype may contribute to these changes. Other growth factors like TNFα (data not shown) might also contribute to changes in PAI-1 expression but might be independent of the 4G/5G PAI-1 polymorphism.

The central position of IL-1β was demonstrated in cultured ECs. Using Firefly luciferase reporter plasmid containing the 4G site in the proximal PAI-1 promoter, our data indicate that IL-1β enhanced *PAI-1* expression in ECs with the 4G4G genotype, similar to earlier studies using HEPG2 cells (18).

The IL-1β-induced fibrinolytic shutdown in the 4G4G genotype was further aggravated by IL-1’s impairment of tPA expression in ECs. PAI-1 upregulation and tPA downregulation in the 4G4G after IL-1β caused “fibrinolytic EC paralysis.”

During COVID-19, the transcription factor KLF2, a master regulator of vascular homeostasis, is downregulated in ECs (45) and was associated with COVID-19-associated EC dysfunction (24). We confirm reports by others that IL-1β decreased *KLF2* gene expression in ECs, with the most robust reduction observed in ECs with the 4G4G but not the 5G5G genotype (**Figure 4D**).

It was speculated that drugs like atorvastatin that can induce KLF2 expression would improve EC functions of COVID-19-damaged ECs due to their anti-inflammatory and anti-thrombotic functions. Our data indicate that the most potent suppression of KLF2 was observed in ECs with 4G4G. Therefore, patients with this genotype might profit from atorvastatin. KLF2 can suppress the transcriptional activity of NFκB (46) and inhibit monocyte activation (47), resulting in an anti-inflammatory status. This reverse expression pattern between the transcription factors was not observed in COVID-19 PBMCs. Spearman analysis indicated that NFκB, not KLF2, was linked to PAI-1 expression. The transcriptional activity of NFκB and KLF2 activity is antagonistically regulated in HUVECs (48). In ECs, the induction of proinflammatory cytokines reduces the expression of KLF2. It is important to note that the activities of NFκB and KLF2 are regulated at the transcriptional level and can also be increased at the protein level (48).

Plasminogen is cleaved by MMPs (MMP-2, -7, -9), elastase, or cathepsin D generating kringle-containing fragments such as angiostatin K1-3, K1-4, and K1-5 (28). The Plg fragment K1-3 is generated by active plasmin and MMP3/7. Together with increases in active plasmin, we found more Plg fragment K1-3 in supernatants of 5G5G than in 4G4G EC cultures after IL-1β stimulation. K1-3 has anti-angiogenic properties (49). Further studies will determine whether K1-3 generation during SARS-CoV-2 infection might contribute to delayed tissue regeneration after inflammation.

Like PAI-1, IL-1 is implicated in the pathogenesis of inflammatory diseases, including arthritis, atherosclerosis, and inflammatory bowel disease, where anti-IL-1 treatments are considered (50). Although IL-1 is regarded as a critical driver of hyperinflammation and cytokine storm (51), studies to block IL-1 (Anakinra, the monoclonal antibody (mAb) canakinumab, or the soluble IL-1 trap rilonacept) were shut down early as the treatment groups did not show clinical improvements. Results of Anakinra (an Ab against the IL-1 receptor alpha) on the improvement of acute respiratory distress syndrome in COVID-19 patients vary from showing no effect (52) to being effective in a subgroup of patients with increased soluble uPAR (s-uPAR) levels (32, 53). We are the first to report a link between s-uPAR release from IL-1-stimulated ECs or sera from COVID-19 patients in the complicated phase. Circulating IL-1β and s-uPAR levels were high in the 5G5G COVID-19 patient group. Further studies will be necessary to determine whether COVID-19 patients carrying the 5G5G, but not 4G4G genotype, or patients with other IL-1β-related inflammatory diseases might benefit from drugs blocking IL-1 signaling.

In conclusion, combining clinical evidence and *in vitro* studies, we identify the 5G5G genotype of the 4G5G polymorphism as a risk factor for inflammation-induced endothelial dysfunction combined with the overactivation of the fibrinolytic system.

## Methods

### Study population and data collection

The inclusion criteria were adult patients older than 18 years, in- and outpatients, patients admitted to Juntendo University with a positive PCR or rapid antigen test for SARS-CoV-2, availability of basic medical information, including ethnicity, patient history, initial blood laboratory data, and outcome data.

The Japanese study population included adults admitted to Juntendo University. All study participants recruited between March 2020 and February 2021 gave informed consent to anonymize their clinical data. The inclusion criteria for COVID-19 cases, clinical phase inclusion criteria, and comorbidity criteria were described earlier (19). We used the criteria to determine the complicated COVID-19 phase according to LEOSS criteria (19): need for new oxygen supplementation due to clinical deterioration, oxygen saturation (SO2) at room air < 90%, partial pressure of oxygen (PaO2) at room air < 70 mmHg, clinically meaningful increase of oxygen supplementation compared to prior oxygen home therapy, an increase of aspartate aminotransferase (AST) or alanine aminotransferase (ALT) > 5 × ULN (upper limit of normal), new cardiac arrhythmia, new pericardial effusion > 1 cm or new heart failure with pulmonary edema, congestive hepatopathy or peripheral edema, catecholamine therapy, life-threatening cardiac arrhythmia, liver failure with an INR > 3.5 (Quick < 50%), a qSOFA score of ≥ 2 or acute renal failure with the need of dialysis.

Opt-out samples of COVID-19 subjects were stored at the Biobank of Juntendo University after obtaining informed consent. Peripheral blood mononuclear cells (PBMCs) and sera were collected and stored from randomly chosen patients. After removing duplicates, missing diagnosis information, and dead patients, 46 COVID-19 patients at diagnosis with clinical data, stored blood sera, and PBMC cells were available for analysis. Clinical data of the selected patients and protein sample data were part of a more extensive study published in (19). We assessed matching quality using standardized mean differences for outcome analysis (biomarker analysis).

### Patient sample analysis

Blood Laboratory data. Blood examinations included the coagulation/fibrinolysis factors D-dimer (≤1 ug/mL), INR (<1.25), fibrinogen (≤400 mg/dL), and platelet counts (120,000–450,000/uL) as well as the inflammatory factors CRP (≤3 mg/L), IL-6 (≤1.8 pg/mL), WBC (4000–8000/uL), and neutrophils (2000–8900/uL). Average values are given in brackets. The investigators and laboratory staff did not know the clinical status or genotypes of the COVID-19 patients during the sample analysis.

### Quantitative PCR

RNA isolation and cDNA extraction have been described previously (54). The qPCR determined the mRNA expression levels using the QuantStudio 3 real-time PCR system (Applied Biosystems, USA) with the TB Green Premix Ex Taq II (TaKaRa, #RR820A). Gene expression was determined by comparative SYBR green qPCR using an Applied Biosystems StepOnePlus Real-time qPCR. We calculated the relative mRNA expression for all genes and mRNAs using the 2-ΔΔCt method. Each qPCR experiment was performed in triplicate and independently repeated two times. Unless otherwise mentioned, gene expression was normalized for all qPCR results to b-actin mRNA expression. The respective forward and reverse primers are listed below:

β-Actin: 5`-CCAACCGCGAGAAGATGA -3`; 5`-CCAGAGGCGTACAGGGATAG -3`
TGF-β: 5`-TGACGTCACTGGAGTTGTACGG-3`; 5`-GGTTCATGTCATGGATGGTGC -3`
IL-6: 5`-CACATTCCTGGTTGCTGGA-3`; 5`-CAGCTTCCACGTCTTCTTGA -3`
KLF2: 5`- CATCTGAAGGCGCATCTG-3`; 5`-CGTGTGCTTTCGGTAGTGG -3`
IL1β: 5`-GCGTTTGAGTCAGCAAAGAAGT-3`; 5`-CATGGAGTGGGCCATAGCTT-3`
IFNγ: 5`-TCTTGGCTTTTCAGCTCTGC-3`; 5`-TTTCTGTCACTCTCCTCTTTCC-3`
NFκB1: 5`-GCTTAGGAGGGAGAGCCCA-3`; 5`-TGCCATTCTGAAGCTGGTGG-3`
PAI-1: 5`-AAGGCACCTCTGAGAACTTCA -3`; 5`-CCCAGGACTAGGCAGGTG-3`
tPA 5`- GCTACGGCAAGCATGAGG-3` 5`-ATGGGTACAGTCTGACATGAGC-3`
uPA 5`-TTGCTCACCACAACGACATT-3` 5`-GGCAGGCAGATGGTCTGTAT-3`

### Genotype assessment using polymerase chain reaction-restriction fragment length polymorphism (PCR-RFLP) analysis

Genomic DNA was extracted from patients’ PBMCs, cultured HUVECs, and amplified by PCR using gene‐specific primers [protocol modified from (55)]. The PAI-1 –675 4G > 5G (rs1799889) polymorphism was detected by PCR-RFLP analysis using forward (5′-CCA ACA GAG GAC TCT TGG TC-3′) and reverse (5′-CAC AGA GAG AGT CTG GCC ACG-3′) primers. The 99-bp product was digested with 1 U BslI for one hour at 55°C. A restriction fragment of 99-bp represented the 4G4G genotype; fragments of 99 bp, 77 bp, and 22 bp represented the 4G5G genotype; and the 77-bp and 22-bp products represented the 5G5G genotype.

To detect the PAI-1 + 43G>A (rs6092) genotype, PCR-RFLP analysis was performed with forward (5′-TGT CTT CCA GAA CGA TTC CTT CAC C-3′) and reverse (5′-GTT GTC AGC TGG AGC ATG GCC-3′) primers. The amplified fragment was 266 bp in length. The PCR products were digested with 2 U PshAI for 16 h at 37°C. The restriction product 266-bp identified the GG genotype; products 266-bp, 172-bp, and 94-bp represented the GA genotype, and the 172-bp and 94-bp products represented the AA genotype.

Genotyping results were confirmed by sequencing 30% of randomly selected samples, followed by DNA sequencing, to validate the RFLP findings at FASMAC, Japan. The concordance of the quality control samples was 100%.

### Western blot analysis in EC culture supernatants

Supernatant samples were centrifuged at 5000 g to remove fibrin and diluted 1:4 in 2x Laemmli sample buffer. Samples (5 µl) were applied on 8% acrylamide gel transferred to a PVDF membrane (Millipore, Immobilon). Then membranes were probed with one of the following primary Abs: uPAR rabbit polyclonal Abs (1 ug/ml (56); Plg rabbit polyclonal Abs (0,5 ug/ml, (57); α2-antiplasmin rabbit polyclonal antibodies (1:1000, #sc73659, Santa Cruz, Dallas, USA). Membranes were stained with secondary Ab conjugated with goat anti-rabbit polyclonal HRP-conjugated, Santa Cruz #sc2004, working dilution working dilution 1:8000) and secondary rabbit anti-hamster HRP-conjugated (1:2000, #ab5745, Abcam, Cambridge, UK). Membranes were developed with the ECL Prime Western blotting reagent (Amersham #RPN2236), and images were taken using the image analyzer ImageQuant LAS 4000 (GE Healthcare). Quantification of the protein bands was performed with ImageJ software. Due to patient sample scarcity, western blot data for IL-1β, uPAR (D2D3), and sVCAM1 in COVID-19 patients were reanalyzed from a data set published on the same patients but without the knowledge of genotypes (15). These data were reanalyzed and sorted according to the patients’ genotypes.

Because Plg is present in the culture medium’s serum, the effects of IL-1β treatment were compared to the respective untreated control supernatants.

Western blots of patients’ serum samples were normalized to the volume of loaded serum, and all the serum markers were measured by other methods (ELISA, activity assay, and clinical data).

### PAI-1 activity by ELISA

Total PAI-1 in serum was measured using the Human PAI-1 Standard ABTS ELISA Development kit (PeproTech, #900-K383). PAI-1 activity samples are derived from a more extensive patient study with a different focus (15). Plasmin/antiplasmin complex (Human PAP ELISA kit; Elabscience #E-EL-H2101), Active TGFβ (BioLegend, #437707), total uPA (American Diagnostics/BioMedica Diagnostics, #897), and uPA/PAI1 complexes (Molecular Innovations, huuPA/PAI-1 KTCX) in sera or EC-supernatants were measured according to the manufacturer’s recommendations.

### HUVEC maintenance and IL-1β treatment

HUVECs (purchased from ATCC, USA) at passages 15-20 were cultured in a culture medium (EGM-2 Endothelial Cell Growth Medium-2 BulletKit, Lonza). Cells were cultured in 1%-Gelatin coated culture plates in an atmosphere with 5%CO_2_ at 37°C. Human Lung Microvascular Endothelial Cells (HMVECs) from LONZA (CC-2527) were kindly provided by Dr. Tamami Hansai, Juntendo University. HMVECs were cultured in the same conditions as HUVECs. BMEC-1 cells (purchased from ATCC, USA) were cultured in Medium 199 (HEPES, no L-glutamine, Sigma) supplemented with 15% fetal bovine serum, 1 mM L-glutamine, 16 units/ml heparin, and 25 mM sodium bicarbonate.

HUVECs/HMVECs were seeded at a density of 1x10^5^ per well using 6-well plates for growth factor stimulation. Cells were kept untreated overnight to ensure cell adherence. Cells were treated with or without recombinant human IL-1β (Peprotech #0606B95). IL-1β was added to cultures at a concentration of 10 ng/ml. After a 24-hour culture period, cells were collected and subjected to RNA isolation. Supernatants of cultures were collected and stored at -80°C until usage in Western blotting.

### Plasmid generation

The basic reporter plasmid pGL4.1[luc2] with the Firefly luciferase reporter gene luc2 (Photinus pyralis) and control plasmid for dual luciferase assay pRL-CMV encoding Renilla luciferase reporter gene (Renilla reniformis) were purchased from Promega (USA).

Human gDNA from our donor gDNA bank, previously identified as homozygous 5G5G or 4G4G, was used as a template for the PAI-1 promotor fragment amplification. Fragments of the PAI-1 5′-flanking region [-811 to +142 relative to the transcription start point (TSP)] (GenBank sequence: X06692.1) were amplified by PCR with KOD polymerase (Toyobo, Japan) using primers:

811F 5′- CGCTAGCCTCGAGGATAAGCTTTTACCATGGTAAC-3′ and
142R 5′- CGAGGCCAGATCTTGATCCTGAAGTTCTCAGAG -3′.

DNA fragments were then subcloned into the Firefly luciferase reporter plasmid pGL4.1[luc2] using NEBuilder HiFi DNA Assembly Cloning Kit (New England Biolabs, USA).

### Cell transfection

HUVEC cells with 4G5G genotype were plated at 70% confluence in EGM-2 media (Lonza, Switzerland) in 24-well plates 24-h before transfection. Cells were co-transfected using Lipofectamine 3000 (Invitrogen, USA) with reporter pGL4.1-4G-[luc2] or 5G plasmid or empty pGL4.1-[luc2] plasmid (500 ng/well) and with of pRL-CMV-null Renilla luciferase plasmid (50 ng/well) as a control for transfection efficiency. Each experiment was performed in triplicate.

### Analysis of transcriptional activation of the 4G/5G PAI-1 promoter using a reporter assay

Twenty-four hours after transfection of HUVECs, human recombinant IL-1β (#200-01B, Peprotech, USA) was added at a final 10 ng/ml concentration. Reporter activities were measured 48 h post-transfection/24 h post-IL-1β-stimulation using a dual-luciferase reporter assay system (Promega, USA). Luciferase activity was normalized to Renilla values to control for differences in transfection efficiency and then standardized to the empty vector control. The values are expressed as means ± SEM.

### Statistical analysis

#### Clinical data statistics

We compared the characteristics of SARS-CoV-2-positive Japanese patients and included some analysis data from healthy donors. We presented continuous and categorical variables as median and n (%). Before predicting the patients’ clinical phases, we organized comorbidities and biomarkers as categorical variables (yes versus no). First, we calculated a contingency matrix by counting the frequencies of patients in different clinical phases and specific variable intervals. Then, the statistical dependence test was performed based on the number of intervals and the obtained frequencies. We considered Fisher’s exact test if the contingency table had a 2 × 2 shape (two-variable intervals). We chose the appropriate test when tables with more than two intervals were analyzed, i.e., we executed the X^2^ test when the frequencies in each cell were at least five. However, in the case of analyzing tables with more intervals and some frequencies below five, Fischer’s exact test simulation was performed using the Monte Carlo approach, available in the R statistical package 3.6.3. This package was used to calculate the Pearson correlation test of the numerical values for all biomarkers. We attributed the mean value for patients with missing data for the BMI. *p* < 0.05 was regarded as significant.

A multivariate odds ratio (OR) and corresponding confidence intervals were presented using the R program for each variable included in regression models. The OR for the 4G/5G genotypes was calculated using the 5G5G genotype as the reference category. The OR for the GG genotypes was estimated using the GA genotype as the reference category. A two-tailed P value < 0.05 was considered statistically significant.

#### Sample analysis statistic

All experiments were performed at least three times. P values < 0.05 were considered statistically significant. Data are presented as mean ± SEM.

Outliers were checked using the ROUT test (Q=0.5%) and removed before group comparison. All data, including outliers, were presented in the actual figure panels. Data distributions were tested for normality using the Shapiro–Wilk normality test. Student’s t-tests (two groups) or ANOVAs (more than two groups) were applied to normally distributed data to evaluate the statistical significance of differences between groups with the Welch test for comparison of groups with heterogeneous variances. A Mann–Whitney (two groups) or a Kruskal–Wallis test (more than two groups) was applied for non-normally distributed data. Spearman’s correlation test was used to test the association between two variables.

The linear correlation test and determination of the Spearman coefficient were applied to estimate the association between tested variables.

Statistical analysis was performed using GraphPad Prism v8.1 (GraphPad Software, USA).

## Data Availability

The datasets for this article are not publicly available due to concerns regarding participant/patient anonymity. Requests to access the datasets should be directed to the corresponding author and can be granted after Juntendo University's ethics committee approval.
